# Microwave-Assisted Synthesis of NiCo_2_O_4_ Double-Shelled Hollow Spheres for High-Performance Sodium Ion Batteries

**DOI:** 10.1007/s40820-017-0164-2

**Published:** 2017-10-31

**Authors:** Xiong Zhang, Yanping Zhou, Bin Luo, Huacheng Zhu, Wei Chu, Kama Huang

**Affiliations:** 1 School of Chemical Engineering, Sichuan University, Chengdu, 610065 People’s Republic of China; 2 School of Electronics and Information Engineering, Sichuan University, Chengdu, 610065 People’s Republic of China

**Keywords:** NiCo_2_O_4_, Double-shelled hollow sphere, Microwave, Sodium ion battery

## Abstract

The ternary transitional metal oxide NiCo_2_O_4_ is a promising anode material for sodium ion batteries due to its high theoretical capacity and superior electrical conductivity. However, its sodium storage capability is severely limited by the sluggish sodiation/desodiation reaction kinetics. Herein, NiCo_2_O_4_ double-shelled hollow spheres were synthesized via a microwave-assisted, fast solvothermal synthetic procedure in a mixture of isopropanol and glycerol, followed by annealing. Isopropanol played a vital role in the precipitation of nickel and cobalt, and the shrinkage of the glycerol quasi-emulsion under heat treatment was responsible for the formation of the double-shelled nanostructure. The as-synthesized product was tested as an anode material in a sodium ion battery, was found to exhibit a high reversible specific capacity of 511 mAh g^−1^ at 100 mA g^−1^, and deliver high capacity retention after 100 cycles.

**Electronic supplementary material:**

The online version of this article (doi:10.1007/s40820-017-0164-2) contains supplementary material, which is available to authorized users.

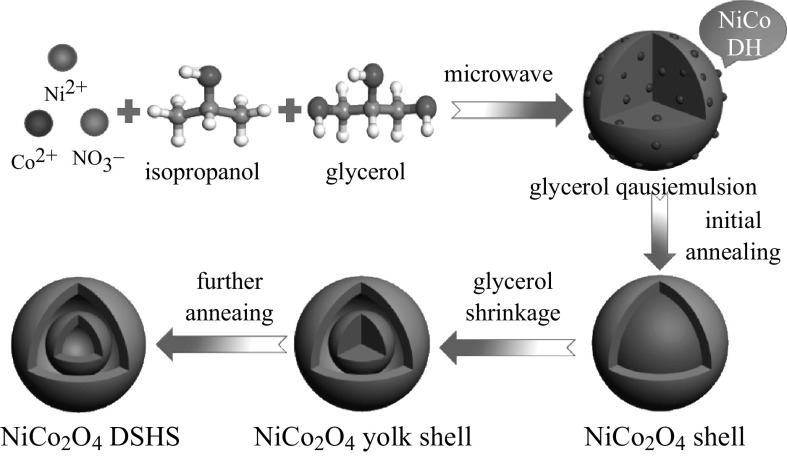

## Highlights

- NiCo_2_O_4_ double-shelled hollow spheres were successfully synthesized via a rapid microwave-assisted solvothermal method in isopropanol with the aid of glycerol.
- The roles of isopropanol, nitrate, glycerol, and the heating rate in the formation of the double shelled hollow spheres were systematically studied.
- The as-synthesized NiCo_2_O_4_ double shelled hollow spheres showed good sodium storage performance with reversible specific capacity of 511 mAh g^−1^ at 100 mA g^−1^.

## Introduction

Presently, due to increasing energy consumption, there is an increasing demand for energy storage materials. Lithium ion batteries (LIBs) offer high energy storage density, long cycling life, and excellent safety properties, thus dominating the market for portable electronic device power sources [1]. However, the depletion of lithium resources and the consequent high cost of lithium hinder the application of LIBs in several emerging areas, such as large-scale grid energy storage [2]. Sodium, another Group I element, is much more abundant and has a much lower cost. As such, sodium ion batteries (SIBs), which have a charging/discharging mechanism similar to that of LIBs, are promising energy storage devices for the future and have received great research attention in the past few years [3]. Nevertheless, the energy storage performance of SIBs is significantly limited by a lack of suitable electrode materials. For example, while graphite is used as the anode material in most commercial LIBs, it is nearly electrochemically inactive with sodium due to the large ionic radius of Na^+^ [4]. Although many other carbonaceous materials have been intensively investigated as anode materials for SIBs, their sodium storage capabilities are too low to meet the demands of practical applications.

Transitional metal oxides have been widely investigated as substitutes for carbonaceous anode materials in LIBs [5]. In particular, ternary transition metal oxides such as NiCo_2_O_4_ are extremely attractive, due to their high theoretical storage capacities (e.g., 890 mAh g^−1^ for NiCo_2_O_4_ compared to 372 mAh g^−1^ for graphite) and superior electrical conductivity (2 orders higher than that of single-component cobalt or nickel oxides) [6]. Theoretically, NiCo_2_O_4_ has equivalent storage capacities for both sodium and lithium. Recently, some work has been reported on the successful application of NiCo_2_O_4_ as an anode material for SIBs [7, 8]. However, due to the sluggish sodiation/desodiation reaction kinetics, as well as the large volume change during the charging/discharging process induced by the large ionic radius of Na^+^, the reported NiCo_2_O_4_ materials exhibit greatly inferior capacities for sodium storage. In order to increase the practical sodium storage capacity of this material, a new strategy to engineer robust nanostructured NiCo_2_O_4_ is urgently needed. One attractive avenue amongst the various approaches is the use of hollow multi-shelled spheres, due to their unique structural features [9–17].

Recently, microwave-assisted nanotechnology has attracted a great deal of research interest, due to the interest in green chemistry in both academia and industry. Microwaves heat the reactants directly via dielectric loss, rather than by heat convection as in the conventional heating method. This unique heating mechanism allows the use of microwaves to greatly enhance the fabrication rate of nanomaterials, thus saving both time and energy. Additionally, nanomaterials synthesized via microwave heating have been widely reported to exhibit excellent performance due to the formation of better dispersed nanoparticles with more uniform size distributions [18].

In this work, we developed a synthesis method for NiCo_2_O_4_ double-shelled hollow spheres, using a fast microwave-assisted solvothermal treatment followed by annealing. Double-shelled hollow nanostructures are beneficial in facilitating a high specific surface area to expose more active materials for reaction, as well as in buffering the volume change during the charging/discharging process. The macropores in the hollow structure can act as a Na^+^ transport system, shortening pathways for the diffusion of Na^+^, thus leading to faster reaction kinetics in hollow nanostructures. The as-synthesized product was further tested as an anode material in SIBs and showed excellent sodium storage capability.

## Experimental

### Materials

All chemical materials were purchased from Aladdin Chemical Corporation and were of analytical grade. The materials were used without further purification.

### Synthesis of NiCo_2_O_4_ Double-Shelled Hollow Spheres

In a typical procedure, 1 mmol of Co(NO_3_)_2_ and 0.5 mmol of Ni(NO_3_)_2_ were dissolved in 80 mL of isopropanol, to which 16 mL of glycerol was added. The mixture was stirred vigorously for 30 min. Subsequently, 60 mL of the mixture was pipetted into a 100-mL vessel and exposed to microwave solvothermal treatment in a microwave hydrothermal reactor (Xianghu, Beijing) at 180 °C for 30 min. To avoid any abnormal increases in pressure due to hot spots during the microwave heating, a ramping procedure was used to raise the temperature from room temperature to 180 °C over 20 min. Finally, the precipitate was collected, washed with ethanol and DI water, dried, and annealed in air at 350 °C for 2 h with a temperature ramping rate of 1 °C min^−1^.

### Characterization of Materials

The crystal structure of the as-prepared samples was examined using X-ray diffraction (XRD, DX-2700, Cu *K*α radiation, *λ* = 1.542 Å). The morphologies and the structural characterization of the products were observed using field emission scanning electron microscopy (FESEM, JEOL, JSM-7500F) and transmission electron microscopy (TEM, Zeiss, Libra200). N_2_ adsorption–desorption measurements were carried out using a NOVA1000e analyzer at 77 K. The pore size distributions of the samples were analyzed using the Barrett Joyner Halenda (BJH) method.

### Electrochemical Measurements

The electrochemical performance of the material was evaluated using CR2032-type coin cells, which were assembled in a glove box filled with highly pure argon gas, with water and oxygen contents of less than 1 ppm. For the fabrication of the working electrode, the NiCo_2_O_4_ material, acetylene black, and the binder polyvinylidene fluoride (PVDF) were mixed in N-methyl-2-pyrrolidinone to form a homogeneous slurry with a weight ratio of 8:1:1, which was then coated on copper foil and finally dried for 12 h in a vacuum oven. The copper foil was cut into rounds with a diameter of 14 mm. The average loading mass of the active material was about 1 mg cm^−2^. Pure sodium foil was used as the counter electrode, and Whatman glass fiber (GF/C) was used as the separator. The electrolyte was 1 M NaClO_4_ in a mixture of ethylene carbonate and propylene carbonate (1:1, *w*/*w*). Galvanostatic charge/discharge tests were conducted using a Neware battery measurement system in the voltage range of 0.01–3.0 V (vs. Na^+^/Na). Cyclic voltammetry (CV) measurements were performed using a CHI 660E electrochemical workstation.

## Results and Discussion

**Fig. 1:**
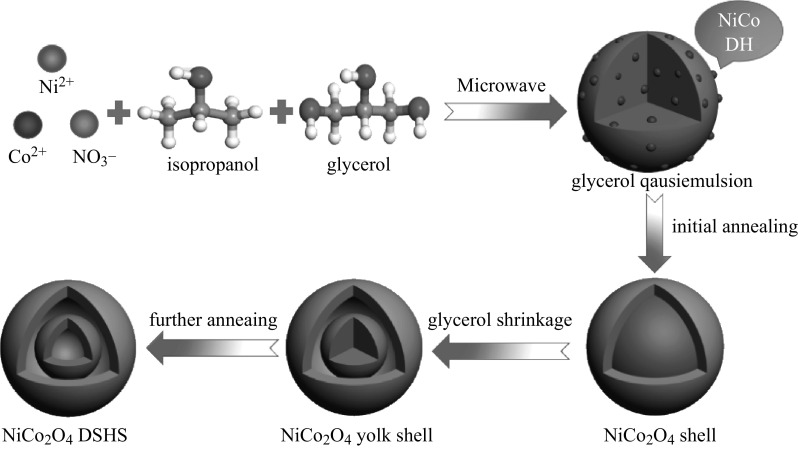
Schematic illustration of the formation process of NiCo_2_O_4_ double-shelled hollow spheres

**Fig. 2:**
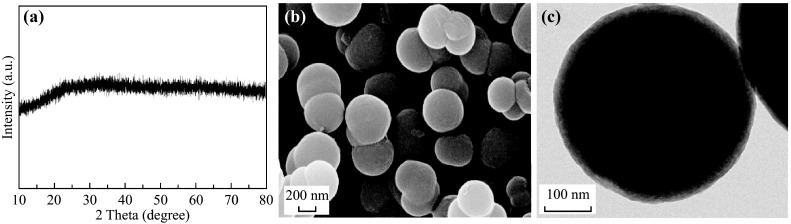
**a** XRD pattern, **b** FESEM image, **c** TEM image for NiCo DH-glycerol composite after microwave solvothermal treatment

The synthesis route of the double-shelled hollow spherical NiCo_2_O_4_ is shown in Fig. 1. At 180 °C, isopropanol and NO_3_^−^ underwent a redox reaction, which is described by the formula: 4(CH_3_)_2_CHOH + NO_3_^−^ = 4CH_3_COCH_3_ + NH_3_ + OH^−^ +2H_2_O, thus releasing hydroxyl ions that precipitated Ni^2+^ and Co^2+^ as Ni–Co double hydroxide (NiCo DH) [19]. Meanwhile, glycerol molecules self-assembled into quasi-emulsions in isopropanol via strong inter-molecular hydrogen bonding under solvothermal conditions, serving as a soft template for the growth of NiCo DH, resulting in NiCo DH-glycerol composites. A control experiment was also conducted to confirm the role that isopropanol and NO_3_^−^ played during the synthesis. When isopropanol was replaced with an equal amount of DI H_2_O, or when NO_3_^−^ was replaced by an equal amount of Cl^−^, no precipitation occurred, indicating that the reduction of NO_3_^−^ to NH_3_·H_2_O by isopropanol and the formation of NiCo DH was the main reason for precipitation, rather than the formation of Ni–Co glycerate as reported by Shen et al. [20]. The XRD spectra (Fig. 2a) of the as-obtained precursor precipitate showed no significant peaks, indicating that the NiCo DH was amorphous. This was probably due to the short reaction time (30 min), which was much shorter than the 6 h required in the conventional heating method. Compared to conventional solvothermal methods for the preparation of metal-glycerate spheres, the microwave-assisted solvothermal method dramatically enhanced the reaction kinetics, thus saving energy and time. The FESEM and TEM images of NiCo DH are shown in Fig. 2b, c. As can be seen, well-dispersed solid spheres of the NiCo DH-glycerol composite with a diameter of about 500 nm were obtained.

**Fig. 3:**
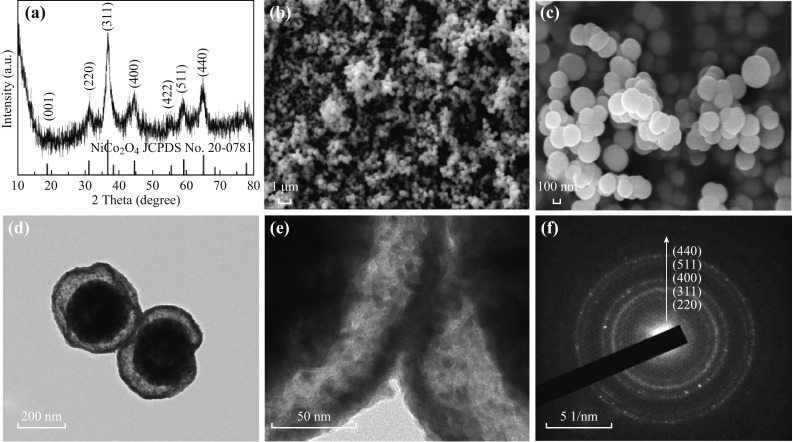
**a** XRD pattern, **b**, **c** FESEM images, **d**, **e** TEM images, **f** SAED pattern of as-synthesized NiCo_2_O_4_ double-shelled hollow spheres

**Fig. 4:**
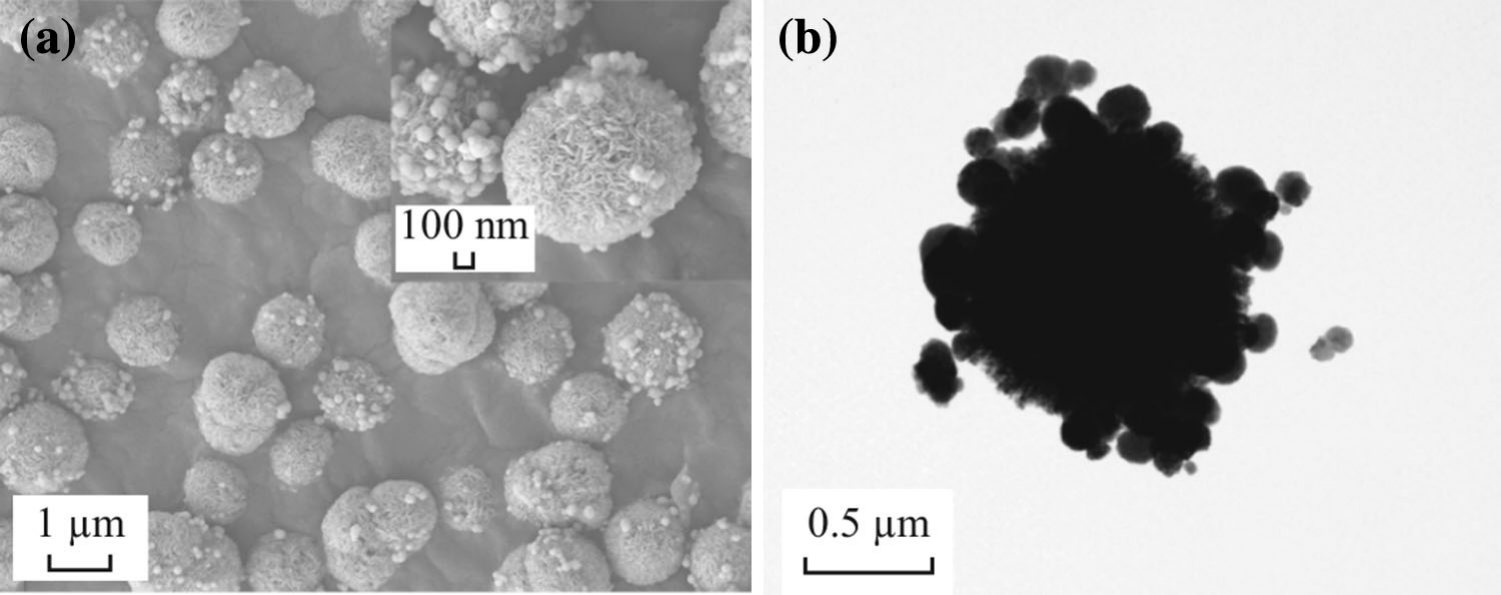
**a** FESEM image, **b** TEM image of the sample prepared without glycerol

The solid precursor spheres were then annealed at 350 °C in air with a temperature ramping rate of 1 °C min^−1^. During the initial annealing process, the large temperature gradient along the radial direction led to the rapid formation of a NiCo_2_O_4_ thin shell on the surface of the NiCo DH-glycerol composite spheres. Thereafter, the glycerol shrank toward the center due to the weight loss caused by oxidation, carrying the embedded NiCo DH with it. After further annealing, double-shelled hollow spherical NiCo_2_O_4_ was formed. The XRD pattern of the as-annealed sample in Fig. 3a showed peaks characteristic of the cubic spinel NiCo_2_O_4_ phase (JCPDS card No. 20-0781). No other phases were detected, indicating that pure crystalline NiCo_2_O_4_ was obtained. Figure 3b, c is the FESEM images of the as-obtained NiCo_2_O_4_, showing spheres with diameters of about 300–400 nm. The NiCo_2_O_4_ spheres were slightly smaller than those of the precursor precipitate, demonstrating the shrinkage of the spheres during annealing. The microstructure of the interior of the sample was further examined using TEM, as shown in Fig. 3d, e. Double-shelled hollow nanostructures are clearly observed, with a ~20 nm outer shell and a ~70 nm inner shell. The selected-area electron diffraction (SAED) pattern can be ascribed to polycrystallinity, and all rings can be indexed to the spinel NiCo_2_O_4_ phase (Fig. 3f). In order to confirm the role of glycerol in facilitating the formation of the hollow structure, a control experiment was carried out in which glycerol was replaced by an equal amount of isopropanol. After annealing at a heating rate of 1 °C min^−1^ (as shown in Fig. 4), solid microspheres with diameters of approximately 1 µm were obtained, demonstrating that glycerol is vital for fabricating the double-shelled hollow nanostructure, as well as to prevent the aggregation of NiCo DH into larger microspheres via a templating effect. Subsequently, to study the effect of the heating rate on the interior structure of the obtained NiCo_2_O_4_ spheres, the NiCo DH-glycerol composites obtained after solvothermal treatment were annealed at heating rates of 5 or 10 °C min^−1^ (TEM images are shown in Fig. S1). The outer shell was found to become thicker as the heating rate increased, and solid spheres were obtained at a heating rate of 10 °C min^−1^. The increase in shell thickness probably occurred because at a higher heating rate, the larger temperature gradient along the radial direction led to more rapid formation of a NiCo_2_O_4_ shell on the surface of the NiCo DH-glycerol composite spheres during the initial annealing process. Such an effect has been described in previous reports [20, 21].

From the N_2_ adsorption/desorption isotherm curve (shown in Fig. S2), the surface area of the as-synthesized double-shelled hollow product was determined to be 30.7 m^2^ g^−1^, with the pore size distribution centered around 6.9 nm. This porous hollow structure is beneficial, both because the high surface area exposes more active materials for reaction during the charging/discharging process and because it could help to buffer the volume change during the sodiation/desodiation process. Thus, this morphology is vital to improve the storage capacity and cycling stability of the electrode.

## Electrochemical Performance

**Fig. 5:**
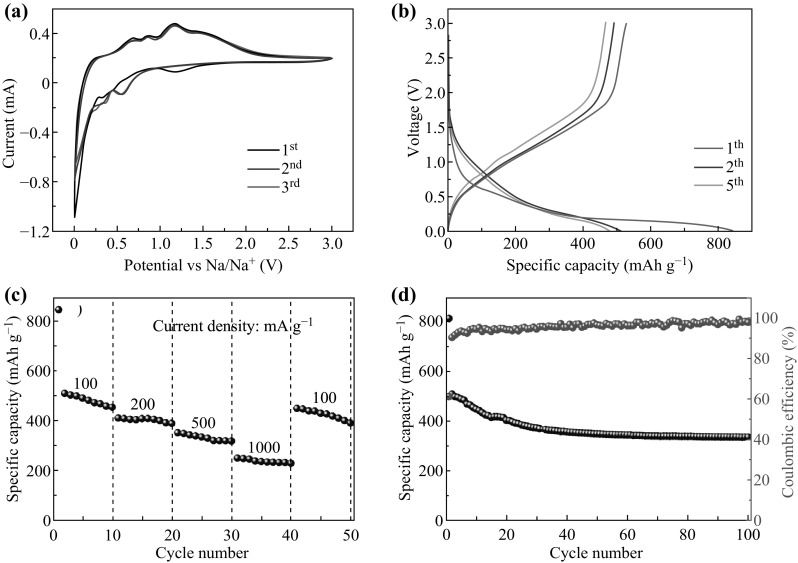
**a** CV curve of the as-synthesized product in the scan rate of 0.2 mV s^−1^. **b** Discharge–charge voltage profiles of as-synthesized product at a current density of 100 mA g^−1^. **c** Rate capacity of as-synthesized product at different current density between 0.01 and 3 V. **d** Cycling stability of as-synthesized product at a current density of 100 mA g^−1^

The electrochemical performance of the as-synthesized NiCo_2_O_4_ double-shelled hollow spheres as an anode material for SIBs was investigated. Figure 5a shows the CV curve at a scan rate of 0.2 mV s^−1^. The peak at 1.2 V in the first cathodic cycle, which disappeared in subsequent cycles, was assigned to the formation of a solid electrolyte interphase layer [22]. Two additional weak peaks at 0.34 and 0.47 V corresponded to the reduction of NiCo_2_O_4_ into metallic Ni and Co, accompanied by the formation of Na_2_O. The peaks at 0.68, 0.85, and 1.17 V in the anodic scan corresponded to the re-oxidation of metallic Ni and Co to NiCo_2_O_4_. Subsequently, the oxidation/reduction peaks for the conversion reaction overlapped and stabilized, indicating the high reversibility of the sodiation/desodiation reaction after the first cycle. Figure 5b shows the typical galvanostatic charge–discharge profiles of the product at a current density of 100 mA g^−1^ during the 1st, 2nd, and 5th cycles. During the first discharge process, the electrode exhibited a long potential plateau at around 0.2 V that disappeared during subsequent cycles. This plateau was ascribed to the phase decomposition of the spinel structure. Similar results have been reported previously [23]. The initial discharge and charge capacities were 814 and 513 mAh g^−1^, respectively, corresponding to a coulombic efficiency of 63%. The irreversibility of the capacity was mainly caused by irreversible SEI film formation reactions as a result of electrolyte decomposition [8]. The discharge and charge curves for the second and the fifth cycles were basically coincident, demonstrating the good cycling stability. The rate capacity of the cells is shown in Fig. 5c. The cell with the NiCo_2_O_4_ electrode delivered high reversible specific capacities of 511, 412, 353, and 251 mAh g^−1^ at 100, 200, 500, and 1000 mA g^−1^, respectively. The capacity recovered to 451 mAh g^−1^ when the current density was switched to 100 mA g^−1^, which was close to the value obtained during the initial cycle, demonstrating the good electrochemical reversibility and structural stability during the charge/discharge process. The cycling performance of the NiCo_2_O_4_ double-shelled hollow spheres at a current density of 100 mA g^−1^ is shown in Fig. 5d. The coulombic efficiency of the cell increases significantly upon cycling, eventually reaching about 98%, illustrating that the charge/discharge process was highly reversible. After 100 continuous cycles, a high reversible discharge capacity of 341 mAh g^−1^ was retained, which corresponded to retention of 66% of the second cycle discharge capacity. The sodium storage properties achieved in the present study were superior to those of many reported NiCo_2_O_4_ materials [7, 24–26] (as shown in Table S1). Clearly, the unique double-shelled hollow sphere structure of NiCo_2_O_4_ played an important role in its excellent electrochemical performance. Specifically, the large surface area and the porous structure were beneficial for promoting Na-ion permeation and the electrochemical reaction between the electrode material and electrolyte, resulting in a high-specific capacity and outstanding rate performance. In addition, the hollow structure can serve as a buffer to resist volume change during the sodiation/desodiation process. As a result, this special structure can endow outstanding cycling stability.

## Conclusions

In summary, a microwave-assisted fast solvothermal synthetic procedure for NiCo_2_O_4_ double-shelled hollow spheres in the presence of isopropanol and glycerol was developed. Both isopropanol and glycerol played a vital role in the synthetic procedure. The as-synthesized product exhibited excellent sodium storage performance when tested as an anode material in SIBs.

## Electronic supplementary material

Below is the link to the electronic supplementary material.

Supplementary material 1 (PDF 681 kb)
