# Supplementary material for: Microwave-Assisted Synthesis of NiCo2O4 Double-Shelled Hollow Spheres for High-Performance Sodium Ion Batteries
Source: Nanomicro Lett. 2017 Oct 31;10(1):13. doi: 10.1007/s40820-017-0164-2 (PMC6199058; doi:10.1007/s40820-017-0164-2)
Supplement: Supplementary file 1 — Supplementary material 1 (PDF 681 kb) [file 40820_2017_164_MOESM1_ESM.pdf]

Supporting Information for

# Microwave Assisted Synthesis of $NiCo_2O_4$ Double-Shelled Hollow Spheres for High Performance Sodium Ion Batteries

Xiong Zhang[1], Yanping Zhou[2, *], Bin Luo[2], Huacheng Zhu[2], Wei Chu[1, *], Kama Huang[2]

[1]School of Chemical Engineering, Sichuan University, Chengdu 610065, People's Republic of China

[2]School of Electronics and Information Engineering, Sichuan University, Chengdu 610065, People's Republic of China

* Corresponding authors. Email: ypzhou11@scu.edu.cn; chuwei1965@scu.edu.cn

## Figures and Table

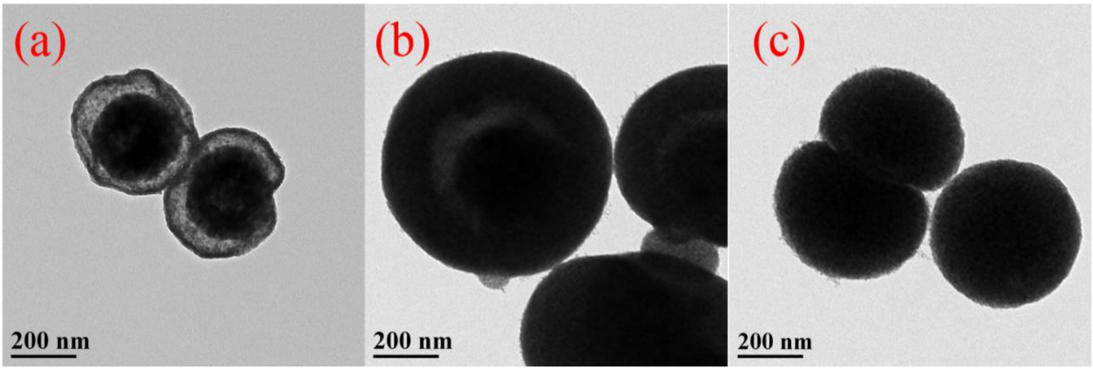


**Fig. S1** The TEM images of $NiCo_2O_4$ obtained at different heating rates **a** 1 °C $min^{-1}$, **b** 5 °C $min^{-1}$, and **c** 10 °C $min^{-1}$

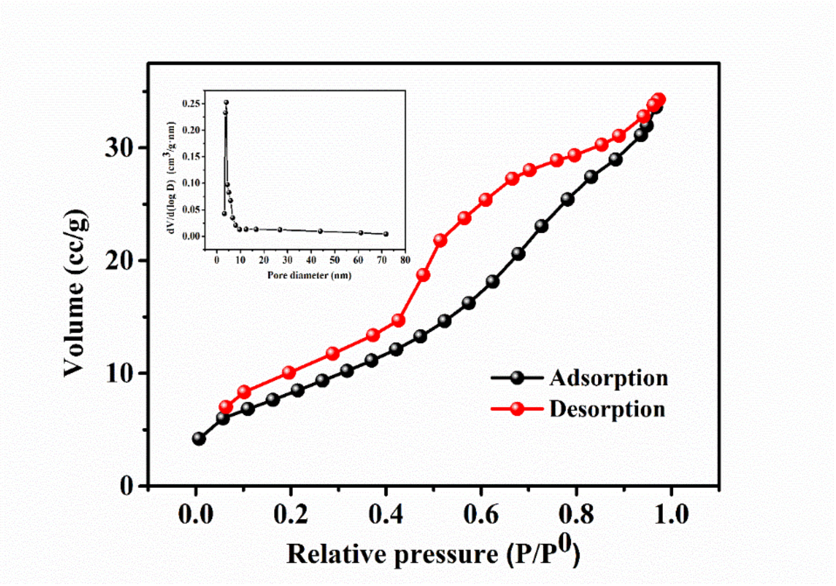


**Fig. S2** $N_2$ adsorption/desorption isotherm and the corresponding pore size distribution curves (insets) of the as-synthesized $NiCo_2O_4$ double-shelled hollow spheres

**Table S1** Sodium storage performance of different $NiCo_2O_4$ electrodes

| Type of material | Capacity ( mAh $g^{-1}$) | Rate capacity ( mAh $g^{-1}$) | Cycling stability ( mAh $g^{-1}$) | Reference |
|---|---|---|---|---|
| $NiCo_2O_4$ double hollow sphere | 814 (at 100 mA $g^{-1}$) | 251 (at 1000 mA $g^{-1}$) | 341 (After 100 cycles at 100 mA $g^{-1}$) | This work |
| $NiCo_2O_4$ hollow nanoboxs | 826 (at 50 mA $g^{-1}$) | ---- | 328 (After 30 cycles at 50 mA $g^{-1}$) | Ref. [7] |
| ---- | 618 (at 0.1 C) | ---- | 200 (After 3 cycles at 0.1 C) | Ref. [24] |
| $NiCo_2O_4$ nanosheet | 690.4 (at 100 mA $g^{-1}$) | 141.8 (at 1000 mA $g^{-1}$) | 203.7 (After 50 cycles at 200 mA $g^{-1}$) | Ref. [25] |
| $NiCo_2O_4$ microrods | 431.1 (at 100 mA $g^{-1}$) | 130.8 (at 1000 mA $g^{-1}$) | ---- | Ref. [26] |
